# Harnessing the hidden genetic diversity for improving multiple abiotic stress tolerance in rice (*Oryza sativa* L.)

**DOI:** 10.1371/journal.pone.0172515

**Published:** 2017-03-09

**Authors:** Jauhar Ali, Jian-Long Xu, Yong-Ming Gao, Xiu-Fang Ma, Li-Jun Meng, Ying Wang, Yun-Long Pang, Yong-Sheng Guan, Mei-Rong Xu, Jastin E. Revilleza, Neil J. Franje, Shao-Chuan Zhou, Zhi-Kang Li

**Affiliations:** 1 International Rice Research Institute (IRRI), Los Baños, Philippines; 2 Institute of Crop Sciences, Chinese Academy of Agricultural Sciences (CAAS), Beijing, China; 3 Shenzhen Institute of Breeding for Innovation/Agricultural Genomics Institute, CAAS, Shenzhen, China; 4 Liaoning Academy of Agricultural Sciences, Shengyang, China; 5 Guangdong Academy of Agricultural Sciences, Guangzhou, China; Clemson University, UNITED STATES

## Abstract

To develop superior rice varieties with improved yield in most rainfed areas of Asia/Africa, we started an introgression-breeding program for simultaneously improving yield and tolerances of multiple abiotic stresses. Using eight BC_1_ populations derived from a widely adaptable recipient and eight donors plus three rounds of phenotypic selection, we developed 496 introgression lines (ILs) with significantly higher yield under drought, salt and/or non-stress conditions in 5 years. Six new varieties were released in the Philippines and Pakistan and many more are being evaluated in multi-location yield trials for releasing in several countries. Marker-facilitated genetic characterization revealed three interesting aspects of the breeding procedure: (1) the donor introgression pattern in specific BC populations was characteristic; (2) introgression frequency in different genomic regions varied considerably, resulting primarily from strong selection for the target traits; and (3) significantly lower heterozygosity was observed in BC progenies selected for drought and salinity tolerance. Applying strong phenotypic selection under abiotic stresses in early segregating generations has major advantages for not only improving multiple abiotic stress tolerance but also achieving quicker homozygosity in early generations. This breeding procedure can be easily adopted by small breeding programs in developing countries to develop high-yielding varieties tolerant of abiotic stresses. The large set of trait-specific ILs can be used for genetic mapping of genes/QTL that affect target and non-target traits and for efficient varietal development by designed QTL pyramiding and genomics-based recurrent selection in our Green Super Rice breeding technology.

## Introduction

As the most important human staple food, approximately 90% of rice is grown and consumed in Asia. The food security threat that shook many Asian countries in 2008 still largely looms as resource-poor farmers face the challenge to produce more rice from lesser and dwindling resources of water, land and costly inputs. In many Asian countries where poverty and rice production are quite interlocked, any effort to alleviate poverty in these regions needs intervention by increasing rice production while keeping the costs of cultivation low and sustainable. Over the past few decades, irrigated rice has been the research focus for enhancing rice productivity by increasing inputs in many Asian countries. Now, irrigated lowlands occupy ~79 million ha that contribute 75% of the rice supply. Rice production in the subtropical regions of western China, Pakistan and northwest India depends largely on supplementary irrigation in the wet seasons. Dry-season irrigated rice areas are mostly concentrated in southern and central China, southern and eastern India and the whole of Southeast Asia, where has recently had a lack of irrigation water at critical crop growth stages in many of these irrigated rice areas. Many countries will be suffering from physical water scarcity by 2025, especially in the wet-season irrigated areas in northern China (2.5 mha), Pakistan (2.1 mha) and northern and central India (8.4 mha) [1]. Likewise, many irrigated paddy lands are now frequented by terminal droughts with a shortage of irrigation water because of global warming. Further increasing the productivity of irrigated rice requires the use of nutrient- and water-use-efficient varieties, which are currently lacking. The complexity of abiotic stresses increases multi-fold as we move to rainfed areas, which have received much less research investment with fewer research products to test.

The detrimental effects of saline soils on rice productivity vary considerably, especially when they often become compounded by mineral deficiencies (Zn, P) and toxicities (Fe, Al, organic acids), submergence (both coastal saline water and river flooding), deep water and drought [2]. Further, these stresses do vary in magnitude across locations and their interactions over space and time make it very difficult for any cultivar to adapt. Rainfed conditions in Asia are quite complex where multiple stresses frequently prevail and even follow in quick succession within a single cropping period. However, two or more abiotic stresses often co-exist in many rainfed and saline areas, even in a single crop season. For example, in many parts of the rainfed lowland areas of Bangladesh, salinity and submergence frequently occur in the early stage of the rice crop, whereas drought and salinity arrive in the terminal stages of the crop [3, 4]. Meanwhile, we have also been witnessing abrupt climatic changes that often spike day and night temperatures, creating more frequent floods in coastal belts and river plains, severe droughts in favorable rainfed lowlands and water shortages in irrigated fields. Rice production under these conditions is becoming more vulnerable and unsustainable. Therefore, multiple abiotic stress tolerance needs to be addressed at the onset of breeding programs targeting these environments.

Progress has been made in developing lines tolerant of a single abiotic stress, particularly for drought, submergence or saline conditions. Breeding for abiotic stress tolerance by a regular pedigree breeding approach has been a long and laborious process with limited success. However, the marker-assisted backcross breeding approach for the transfer of a single major-effect QTL has been used extensively over the past few decades, especially for the transfer of the flooding tolerance gene *SUB1*, salinity tolerance gene *Saltol* and a few drought QTLs [5]. These corrective-breeding technologies have paid rich dividends, especially in the case of the deployment of flood-tolerant Swarna-Sub1 in India and Bangladesh [6]. Most of the rainfed areas in South and Southeast Asia are often visited by multiple abiotic stresses such as flooding, drought and salinity even within the same cropping season near the coastal areas. Therefore, we need to breed new materials that could tolerate more than one abiotic stress and yield high under normal favorable rainfed conditions as well. This is essential as many of the abiotic stress conditions are not a regular occurrence and therefore varieties with abiotic stress tolerance must yield high under normal favorable conditions. Our previous backcross breeding efforts had clearly demonstrated that sufficient genetic diversity exists in the primary gene pool for improving almost all complex traits, including yield potential and abiotic stress tolerance [7–11]. However, few breeding efforts have been attempted to develop rice varieties with improved yield plus tolerance of two or more abiotic stresses that are more suitable for rainfed conditions. In this article, we would like to demonstrate an improved BC breeding approach that results in a faster breeding cycle to fix breeding lines tolerant of multiple abiotic stresses (drought and salinity) while performing well under normal irrigated conditions. This innovative way of breeding, labeled Green Super Rice (GSR) breeding technology for developing new varieties, is now adopted to breed a large set of materials with improved tolerance of multiple abiotic and biotic stresses. Here, we wish to compare and discuss the different selection schemes within this innovative breeding process based on comprehensive phenotypic data from replicated experiments and SSR marker genotypic data to better understand the breeding method.

## Materials and methods

### Plant materials

The materials in this study comprised eight bulk BC_1_F_2_ populations derived from crosses between Huang-Hua-Zhan (HHZ, the recipient) and eight donors. HHZ is a high-yielding and widely adaptable Chinese *indica* variety from Guangdong Province of China and showed moderate tolerance to salt and drought stresses. The eight *indica* donors are from China, India, the Philippines and Vietnam (Table 1). The selection of eight donors had nothing to do with either their abiotic stress tolerance or their performance under irrigated conditions, as we had sufficient evidence that any unrelated rice lines carry useful “hidden” genetic variation for complex traits such as abiotic stress tolerance [7]. HHZ was crossed with each of the donors in the 2007 early season and the F_1_s were backcrossed to HHZ once in the 2007 late season. Seeds from more than 25 segregating BC_1_F_1_ plants of each cross were bulk harvested without selection to form a single BC_1_F_2_ population. The eight BC_1_F_2_ bulk populations derived from eight donor introgression lines (ILs, in parentheses) in the HHZ background are HHZ5 (OM1723), HHZ8 (Phalguna), HHZ9 (IR50), HHZ11 (IR64), HHZ12 (Teqing), HHZ15 (PSBRc66), HHZ17 (CDR22) and HHZ19 (PSBRc28). The materials also included 496 BC_1_F_5_ ILs developed from the eight BC_1_ populations after two rounds of phenotypic selection for high yield under normal irrigated conditions and for abiotic stress tolerance under stress conditions, and five standard check varieties described below. These ILs ranged from 45 ILs from population HHZ/PSBRc66 to 76 ILs from population HHZ/OM1723 (Table 1). All lines were collected from the International Rice Research Institute (IRRI).

**Table 1:** The numbers of selected BC_1_F_2_ and BC_1_F_3_ plants from two rounds of selection for yield, drought tolerance (DT) and seedling salt tolerance (ST) from eight Huang-Hua-Zhan (HHZ, the recipient) BC_1_ populations.

	Donor		BC_1_F_2_				BC_1_F_3_ ^a^			
Population	Name	Origin	Yield	DT	ST	Total	Yield	DT	ST	Total
HHZ5	OM1723	Vietnam	11	21	15	47	26(12)	22(17)	28(11)	76
HHZ8	Phalguna	India	9	13	15	37	15(5)	15(5)	26(8)	56
HHZ9	IR50	Philippines	5	15	15	35	18(6)	13(5)	30(9)	61
HHZ11	IR64	Philippines	11	10	15	36	26(12)	7(2)	24(7)	57
HHZ12	Teqing	China	31	15	15	61	21(13)	18(8)	27(8)	66
HHZ15	PSBRc66	Philippines	8	7	15	30	21(7)	3(1)	21(3)	45
HHZ17	CDR22	India	16	12	15	43	15(11)	18(6)	35(12)	68
HHZ19	PSBRc28	Philippines	9	16	15	40	21(7)	9(3)	37(15)	67
Total			100	109	120	329	163(73)	105(47)	228(73)	**496**

^a^ The numbers in parentheses are those BC_1_F_3_ plants selected from the second round of selection.

### Selection schemes for improving abiotic stress tolerance and yield

**Fig 1:**
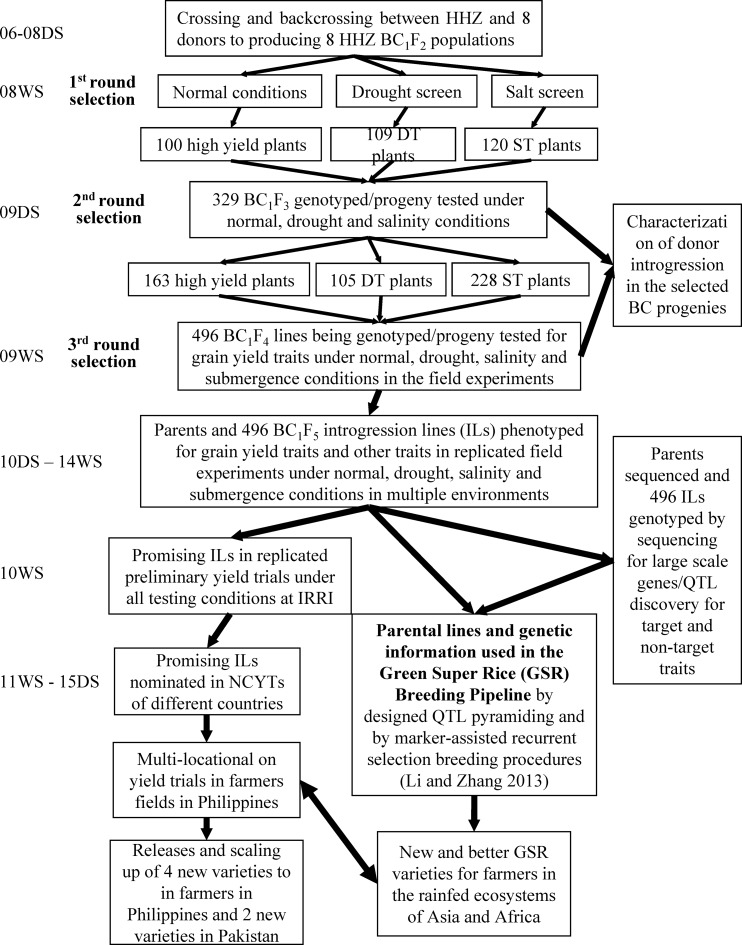
The BC breeding procedure for improving multiple complex traits in rice in which WS and DS represent the wet and dry seasons, and DT and ST represent drought and salt tolerance

IRRI granted permission of field experiments. Fig 1 shows the phenotypic selection schemes of the BC_1_F_2_ bulk populations to develop ILs with improved yield and tolerance of one or more abiotic stresses, which included one round of single plant selection for higher grain yield under irrigated conditions and for single abiotic stress tolerance in the BC_1_F_2_ generation, followed by one round of progeny testing of all selected BC_1_F_3_ lines across different stress and non-stress conditions, as described below.

#### Screen for drought tolerance (DT)

Drought trials were conducted on the Upland Rice Farms of the IRRI Experiment Station from WS2008 onward. Seeds of each BC_1_F_2_ population were sown on a seedling bed and 588 21-day-old seedlings of each BC_1_F_2_ population were transplanted into a 42-row plot with one row of HHZ inserted every 10 rows in the flooded field of the upland field as the checks. Then, irrigation was maintained for 1 month, the field was drained and irrigation was withheld completely till harvest. Thus, the plant materials were typically subjected to moderate (in the wet season, WS) to severe (in the dry season, DS) drought at the reproductive stage depending on the rainfall during the testing seasons (S1 Table). During the periods of drought stress, the water levels of the fields were monitored daily in the root zone by tensiometers (EM50 Decagon Devices Inc., Pullman, WA, USA), which had special sensors to monitor the drought stress, especially the severe drought conditions near the root zone during the DS.

#### Screen for salinity tolerance (ST)

Seeds of the eight BC_1_F_2_ bulk populations in two separate batches were screened for ST at the seedling stage with SNAP culture solution at 18 dS m^-1^ under screenhouse conditions. Surface-sterilized seeds of individual BC_1_F_2_ bulks were germinated in Petri dishes at 30°C for 48 h. Each Styrofoam float was specially made with a nylon mesh bottom to accommodate two germinated seeds per hole and floating on distilled water in plastic containers [12]. For each of the BC_1_F_2_ bulks, 520 seedlings were screened along with HHZ, FL478 (tolerant check) and IR29 (susceptible check). Three days after the seedlings were established, distilled water was replaced by salinized nutrient solution with EC of 6 dS m^-1^ [13]. Three days later, the salinity level was raised to 12 dS m^-1^ by adding NaCl to the SNAP solution. This level was maintained for up to 2 weeks. Then, the EC of the nutrient solution was raised to 18 dS m^-1^ for 1 week. The solution was replenished every 5–8 days and pH adjusted daily to 5.0. The screening was carried out up to 24 d from the initial salinization until the susceptible check HHZ was dead. The surviving BC_1_F_2_ plants were then transferred to the screenhouse to produce their BC_1_F_3_ seeds.

#### Selection for high yield (HY) under normal irrigated conditions

Seeds of each BC_1_F_2_ population were sown in a seedling nursery and 588 plants were transplanted into the same 42-row plot with one row of HHZ inserted in every 10 rows in the irrigated field as the check on the IRRI farm. The field was irrigated regularly and managed with the standard crop management practices. At maturity, promising HY plants free of disease from each population were visually selected based on their overall superior agronomic performance based on a direct comparison with the nearest check, HHZ.

#### Second-round selection by progeny testing across both stress and non-stress conditions

First-round selection of the eight BC_1_F_2_ populations under abiotic stresses resulted in a total of 329 BC_1_F_3_ ILs (Fig 1 and Table 1). These included 109 DT ILs, 120 ST ILs and 100 high-yielding ILs selected under normal irrigated conditions. In the second round of progeny testing, each of the BC_1_F_3_ lines obtained from the first-round selections was planted into single-row plots with 15 plants in each row across all three testing conditions: normal irrigated, drought stressed and salt stressed, with HHZ inserted every 10 rows as the check. Selection was based first on line performance when compared with the nearest check; then, one to three best plants were individually selected from each of the promising lines that visually performed better than the check. Thus, the second-round selection across multiple environments resulted in 496 selections, including 163 HY plants from 73 BC_1_F_3_ lines under normal irrigation, 228 ST plants from 73 BC_1_F_3_ lines under salinity stress and 105 DT plants from 54 BC_1_F_3_ lines under drought stress. Thus, all 496-selected ILs could be grouped into nine selection schemes based on the target traits in the two rounds of selection.

### Replicated phenotyping of the BC_1_F_5_ HHZ ILs for yield and related traits under stress and non-stress conditions

To evaluate the grain yield and overall performance of the 496 HHZ BC_1_F_5_ ILs against the standard check varieties in order to identify superior lines to be nominated to national cooperative yield trials (NCYT) in different countries, the 496 BC_1_F_5_ HHZ ILs and five standard check varieties were evaluated for their yield performance in replicated trials of the same experimental design under irrigated non-stress, drought and salinity conditions during the 2012 dry season in the Philippines. The five checks included HHZ, two irrigated checks (PSB Rc82 and NSIC Rc222), a rainfed check (NSIC Rc192) and a DT check (IR74371-70-1-1). An additional ST check, NSIC Rc184, was used only in the salinity experiment. Under irrigated non-stress conditions, seeds of the 496 HHZ BC_1_F_5_ ILs and two other checks (PSB Rc82 and NSIC Rc222) were sown in the seedling nursery on Dec. 20, 2011, and 25-day-old seedlings of each IL or check were transplanted into a two-row plot with 12 plants per row in the irrigated field of the IRRI lowland experimental farm on Jan. 9, 2012. During the whole growth period, 5 cm of standing water was maintained in the field until 2 weeks before harvest, and the drought stress of the experiment began at 30 days after transplanting at the peak tillering stage before panicle initiation (PI) by draining the field and withholding irrigation until maturity. The 496 ILs and five checks were randomly assigned in the field using a randomized complete block design (RCBD) in two replications with spacing of 15 x 25 cm. In addition, HHZ, being the recipient parent, was inserted every 10 plots within each replicate for effective comparison. The irrigated condition was on a farm. The field was managed with the standard management practices applied with 160:60:60 kg of fertilizer according to the N:P:K ratio. Nitrogen was applied to irrigated plots at 160 kg ha^-1^ with five splits: 30 kg ha^-1^ basal; 30 kg ha^-1^ at 18 days after transplanting (DAT), to promote tillering; another 30 kg ha^-1^ at 33 and 43 DAT; and, finally, 40 kg ha^-1^ at 64 DAT, which is somewhere around PI, to promote spikelet differentiation. All plots were recorded for their heading date when 50% of the plants started heading, and five representative plants within each plot were measured for their plant height and harvested at maturity for measuring grain yield (g/plant) and yield-related traits.

For the drought experiment, all ILs, HHZ and the checks were transplanted into the upland field of the IRRI experimental farm with an RCBD in two replications with spacing of 15 x 25 cm. Seeds of the 496 HHZ BC_1_F_5_ ILs, HHZ and four other checks were sown in the seedling nursery on Dec. 27, 2011, and 20-day-old seedlings of each IL or check were transplanted into a two-row plot with 12 plants per row in the drought upland field of the IRRI experimental farm on Jan. 16, 2012. For the drought plots, 30:30:30 kg NPK fertilizer was applied for basal and 30 kg ha^-1^ of nitrogen was applied at 18 and 33 DAT to fully match growth needs in nitrogen. The drought stress began at 30 days after transplanting by draining the field and withholding the irrigation. The severity of drought was monitored by data logger TM50 (Decagon Devices Inc., Pullman, WA, USA) and the soil water tension ranged from 100 to 300 kPa during the drought stress period. The average rainfall on the upland farm during the application of drought until harvest was 2.15 mm day^-1^, with total rainfall of 155.4 mm.

The salinity experiment was conducted in a natural coastal saline farmer’s field in Infanta, Quezon, Philippines. An ST check, NSIC Rc 184, was also included in this trial. The experiment was laid out in an RCBD in two replications with spacing of 15 x 25 cm. Seeding of the HHZ ILs and checks was carried out on Dec. 27, 2011, and 23-day-old seedlings were transplanted into the field on Jan. 19, 2012. Fertilizer of 120:40:40 kg NPK was applied with 50% of N and 100% of P and K as basal and 25% of N at active tillering (18 DAT) and 25% of N (33 DAT) near flowering to fully match growth needs. The data logger TM50 (Decagon Devices Inc., Pullman, WA, USA) was used to monitor salinity with sensors placed at 10 cm of the root zone. Salinity ranged from 6 to 14 dS m^-1^ during the entire growth period from seeding to harvest depending upon the rainfall and high tides.

### Progeny testing of HHZ ILs

After one more round of progeny testing and two consecutive seasons of preliminary yield trials across four testing conditions (normal, drought, salinity and submergence), many BC_1_F_5_ ILs were nominated to NCYTs of different ecosystems in the target countries.

### Genotyping of HHZ ILs

A total of 625 SSR markers from Cornell University [14] were used to survey the polymorphisms between HHZ and the donors and 397 polymorphic SSR markers were used to genotype the 329 bulk BC_1_F_3_ ILs and the final 496 bulk BC_1_F_4_ ILs obtained from the first round and second round of selection to recover the genotypes of their original selected BC_1_F_2_ and BC_1_F_3_ plants. The same set of polymorphic SSR markers was used to genotype each of the BC progenies, ranging from 49 markers in population HHZ12 to 91 markers in population HHZ9.

### Data analyses

Analysis of variance (ANOVA) was used to compare the yield differences among the testing environments (E), among different ILs (genotypes) within each population, ILs from different populations, among ILs from different selection schemes and G x E interactions of the 496 HHZ BC_1_F_5_ ILs, and to compare the yield differences between the ILs and checks evaluated in different testing environments. Because all ILs had gone through two rounds of phenotypic selection for the three target traits, there were nine different selection schemes. For example, 72 ILs resulted from two rounds of phenotypic selection for high yield designated as YY, 33 ILs from selection for DT in first-round selection and for high yield in second-round selection, designated as DY, and so on. Thus, ANOVA was also performed to compare the efficiencies of different selection schemes in improving the three target traits based on the data from replicated progeny testing. Here, selection efficiency is defined as the number of progenies showing significant improvement in target traits selected by different selection schemes. The genotypic frequencies of each selected HHZ IL were calculated from all polymorphic SSR markers used in genotyping to characterize the introgression patterns in ILs from different populations and from different selection schemes. *X*^*2*^ tests were performed to compare the genotypic and introgression frequencies of the ILs from different populations and from different selection schemes. Finally, we define here breeding efficiency as the number of new varieties released per breeding population (cross) to compare our breeding approach with the empirical pedigree breeding methods under discussion.

## Results

### Results of selection

Table 1 shows the results of the eight HHZ BC_1_ populations in the two rounds of selection. In the first round of selection, only 109 plants in the eight BC_1_F_2_ populations survived and produced seeds under severe drought stress, ranging from 7 plants in population HHZ15 to 21 plants in population HHZ5. Under normal irrigated conditions, 100 HY plants were visually selected based on overall yield performance and plant type, ranging from 5 selections from population HHZ9 to 31 plants from population HHZ12. In the salinity screen, many seedlings from each population survived the stress. After being transferred to the screenhouse, only 15 selected BC_1_F_2_ plants were retained from each population based on their overall performance.

All 329 BC_1_F_2_ plants from the first round of selection were progeny tested across all three test environments, whereas the second round of selection was practiced first to select good BC_1_F_3_ lines under each condition, and then to select one to three best-performing plants from each selected line. Under the drought stress, only 47 BC_1_F_3_ lines performed better than the HHZ check and comparable to drought check i.e. IR74371-70-1-1 from which 105 BC_1_F_4_ plants were selected. Under salt stress, 73 BC_1_F_3_ lines performed better than HHZ, from which 228 BC_1_F_4_ plants were selected. Under normal irrigated conditions, 163 best yield performing BC_1_F_4_ plants were visually selected from 73 lines based on overall phenotypic performance when compared with HHZ. Together, the second round of selection resulted in a total of 496 BC_1_F_4_ plants selected from 213 BC_1_F_3_ lines.

### Yield performances of BC_1_F_5_ progenies from different selection schemes

ANOVA indicated that almost all the sources of the variation were statistically significant, with the testing environments (E) accounting for the largest yield variation of the HHZ ILs, followed by ILs × E interaction within populations, selection schemes within populations and mean yield variation among ILs from different populations (Table 2).

**Table 2:** Analysis of variance for the grain yield performance of 496 BC_1_F_5_ introgression lines selected from eight HHZ populations by nine selection schemes (SS) under three environments (E) during the 2012 dry season.

Source of variation	Df	SS	MS	F	P
Population (P)	7	4173.6	596.2	27.3	1.0E-14
Selection schemes (SS) within P	44	2180.2	49.5	2.3	6.1E-06
ILs within SS	444	14131.1	31.8	1.5	2.9E-07
Testing environments (E)	2	42882.0	21441.0	980.7	1.0E-14
P*E	14	2302.4	164.5	7.5	1.0E-14
SS*E within P	88	3545.9	40.3	1.8	7.0E-06
ILs*E within P	852	22794.8	26.8	1.2	5.3E-04
Replications within E	3	307.7	102.6	4.7	2.9E-03
Error	1324	28947.7	21.9		

Table 3 and S2 Table summarize the yield performance of the 496 HHZ BC_1_F_5_ lines developed from nine selection schemes in the replicated yield trials under normal, drought and saline conditions. Of the nine selection schemes, DS generated most (30.4%) of the selected BC_1_F_3_ lines (N1), followed by YY, YD, SY and SS, whereas the other four produced many fewer progenies. Under normal irrigated conditions, the average yield of the 496 BC_1_F_5_ ILs was the same as that of HHZ. However, different lines within each selection scheme varied considerably in their yield. Of these, 87 ILs (17.5%) had significantly higher yield than HHZ, with an average yield advantage of 30.1%, whereas 97 ILs (19.5%) had significantly lower yield than HHZ, with an average yield disadvantage of 37.4%. Of the nine selection schemes, DD and SS had significantly higher selection efficiency (around 28%) in producing high-yielding progenies under normal conditions, in which selection scheme YY produced ILs with significantly higher mean yield than other selection schemes.

**Table 3:** The mean grain yield (g/plant) performance of BC_1_F_5_ introgression lines (ILs) from different selection schemes (SS) under normal, drought and salinity conditions during the 2012 dry season at IRRI.

Selection scheme	N1^a^	Normal conditions					Drought					Salinity				
		Mean±SD	High-yield ILs		Low-yield ILs		Mean±SD	High-yield ILs		Low-yield ILs		Mean±SD	High-yield ILs		Low-yield ILs	
			N2^b^	Mean±SD^d^	N3^c^	Mean±SD		N2	Mean±SD	N3	Mean±SD		N2	Mean±SD	N3	Mean±SD
YY	72	24.8±5.5	11	34.8±2.3a	15	18.1±1.3	11.7±3.2b	43	13.6±2.0	8	5.8±2.0	17.9±4.2ab	23	22.6	10	11.5
DY	33	23.8±5.1	5	31.6±1.8b	7	17.1±3.0	11.3±3.9b	19	13.7±2.9	5	5.7±2.1	16.0±4.3cd	6	22.4	12	11.6
SY	57	23.5±5.6	10	32.1±2.4ab	15	17.1±2.7	11.1±3.0b	32	13.0±1.9	7	6.0±1.5	16.9±4.4bcd	14	21.9	14	10.7
YD	59	24.3±4.9	7	32.2±2.1ab	11	16.9±2.5	13.1±3.3a	46	14.4±2.6	2	6.9±0.8	18.8±4.6a	25	23.2	8	12.1
DD	24	24.8±5.2	7	31.4±3.1b	6	18.9±0.7	11.7±3.5b	17	13.6±2.3	5	6.5±1.4	15.7±4.4d	7	21.4	10	11.6
SD	20	24.0±4.2	3	31.3±1.7b	3	18.2±1.2	12.0±2.7ab	11	13.8±2.1	0	-	15.5±3.7d	3	21.5	6	11.4
YS	21	24.4±5.2	3	31.9±1.9b	3	16.4±4.3	13.1±3.1a	19	13.6±2.9	0	-	18.2±5.5ab	10	22.6	6	11.5
DS	151	24.3±5.4	26	31.6±2.5b	27	16.3±2.9	11.6±3.5b	94	13.6±2.1	23	6.1±2.0	16.7±4.7c	35	23.6	38	11.2
SS	54	25.5±5.1	15	30.9±1.5b	10	17.3±1.6	11.9±3.1ab	35	13.7±2.0	8	7.0±0.5	18.2±5.1ab	14	24.5	7	10.6
Mean	496	24.4	87	32	97	15.4	11.8	316	13.7	58	6.2	17.2	137	23	111	11.3
HHZ (CK)		24.6					9.4c					16.7c				

^a^ N1 = the total number of lines selected from different selection schemes.

^b^ N2 = the number of selected lines with significantly higher grain yield than HHZ under the testing conditions.

^c^ N3 = the number of selected lines with significantly lower grain yield than HHZ under the testing conditions.

^d^ Different letters indicate statistically significant difference in mean trait values at P ≤0.05, based on Duncan tests.

Under the drought stress, the 496 HHZ BC_1_F_5_ ILs suffered an average yield loss of 51.6%, whereas the check (HHZ) had a yield loss of 61.8%. Of these ILs, 316 (63.7%) had significantly higher yield than HHZ, with a mean yield advantage of 45.7% under drought, whereas only 58 ILs (11.7%) had significantly lower yield than HHZ, with a mean yield disadvantage of 34.0%. Selection schemes YS, YD and DD had significantly higher efficiency than the others in producing DT progenies (Table 3).

Under the salt stress, the 496 HHZ BC_1_F_5_ ILs suffered a significant mean yield loss of 29.5%, whereas HHZ had a yield loss of 32.1%. Of these ILs, 137 (27.6%) had significantly higher yield than HHZ, with a mean yield advantage of 37.7%, whereas 111 ILs (22.4%) had significantly lower yield than HHZ, with a mean yield disadvantage of 32.3%. Again, selection schemes YD and YS showed significantly higher selection efficiency, above 40%, whereas others had lower efficiency in producing high-yielding progenies under salt stress. Interestingly, selection schemes YD, YS and SS produced ILs with significantly higher yield under salt stress (Table 3).

### Comparison of different selection schemes in producing progenies with the best yield performance under different testing conditions

In any breeding program, breeders are particularly interested in the best yield performers because only the best ones are most likely to be released as new varieties. Table 4 shows the yield performance of the top 14 ILs for best yield performance and five checks under each of the testing conditions. The 14 best lines under normal conditions had great yield advantages over HHZ, ranging from 35.4% to 56.9%, four of which had significantly higher yield than the best check, NSIC Rc222, by 10.1‒14.5%. Interestingly, all these high-yielding lines, except for two, had significantly higher yield than HHZ by 14.9‒103.2% under drought conditions, four of which had significantly higher grain yield than the best check, NSIC Rc222, by 22.1‒56.6%. Of these, HHZ17-D1-S2, HHZ11-Y10-Y2, HHZ17-D4-S3 and HHZ5-Y2-Y1 showed significantly higher grain yield under all three conditions. HHZ17-D1-S2 had the highest mean grain yield of 38.6 g/plant, followed closely by HHZ11-Y10-Y2 (37.6 g/plant) and HHZ5-D8-D1 (37.3 g/plant). Eight also had significantly higher yield than HHZ under salt stress, but three lines showed significantly lower yield than HHZ under salt stress. Six of these high yielders resulted from selection scheme YY, four from DS, two from YD and the remaining two were from SY and DD, indicating that the number of best yield performing lines under normal conditions was associated with selection under either normal or drought conditions.

**Table 4:** Yield performance of the 14 best-performing lines under each of normal, drought and salt conditions, respectively, during the 2012 dry season at IRRI.

Top 14 high-yielding lines^a^					Top 14 drought-tolerant lines					Top 14 salt-tolerant lines				
Order	Lines ^1^	Normal	Drought	Salinity	Order	Lines	Drought	Normal	Salinity	Order	Lines	Salinity	Normal	Drought
1	HHZ17-D1-S2	38.6**	12.1*	21.8*	1	HHZ19-D12-Y2	23.2***	16.5*	18.0	**1**	***HHZ5-S14-S2***	34.4**	28.1	18.4**
2	HHZ11-Y10-Y2	37.6**	12.9**	22.3*	2	HHZ5-Y4-S2	21.8***	29.0*	17.6	**2**	HHZ8-D10-S3	32.7**	22.2	15.1**
3	HHZ5-D8-D1	37.3**	10.9*	12.5*	3	***HHZ8-Y2-D1***	21.3***	28.6	26.9**	3	HHZ5-S11-S2	32.1**	26.7	6.6*
4	HHZ19-S7-Y3	37.2**	10.8*	17.1	4	HHZ19-D14-S2	20.8***	14.7*	10.8*	**4**	HHZ5-Y3-S1	29.3**	29.0	13.8**
5	HHZ17-Y16-Y3	36.8**	11.0*	10.7*	**5**	HHZ8-Y3-D1	20.0***	29.6*	19.5	5	HHZ12-Y4-D3	29.2**	26.8	17.8**
6	HHZ11-Y11-Y2	36.7**	9.4	12.5*	6	HHZ17-D8-S1	19.6***	22.7	18.2	**6**	HHZ19-Y2-S1	29.1**	17.9*	12.6**
7	***HHZ5-Y1-Y1***	36.2**	10.5	27.9**	7	HHZ5-Y4-D2	19.6***	27.4	19.1	**7**	HHZ5-Y4-D3	28.4**	27.5	18.0**
8	***HHZ17-D4-S3***	36.1**	19.1***	20.7*	**8**	HHZ17-S1-Y1	19.5***	32.9*	20.5*	8	HHZ11-D10-S3	28.0**	18.7*	13.8**
9	HHZ5-Y2-Y1	36.0**	13.8*	21.0*	**9**	HHZ5-Y8-Y2	19.4***	23.5	16.7	9	HHZ5-Y4-Y1	28.0**	28.1	14.2**
10	HHZ17-D4-S1	35.8**	14.9*	16.1	10	HHZ5-Y3-S2	19.3***	29.7*	17.9	10	***HHZ5-Y1-Y1***	27.9**	36.2**	10.5
11	HHZ12-D1-S3	35.3**	11.0*	19.2	11	HHZ11-D10-S4	19.3***	25.1	18.9	11	HHZ17-S7-S2	27.6**	29.9*	12.9**
12	***HHZ5-Y2-Y2***	35.1**	13.1**	26.8**	12	***HHZ17-D4-S3***	19.1**	36.1**	20.7*	12	HHZ11-Y10-D3	27.0**	31.7*	13.4**
13	HHZ8-Y1-D3	33.9*	15.6**	22.7*	13	HHZ17-D8-S2	19.0**	26.7	23.7*	13	***HHZ8-Y2-D1***	26.9**	28.6	21.3***
14	HHZ17-Y13-D1	33.3*	15.0**	20.5*	14	***HHZ5-S14-S2***	18.4**	28.1	34.4**	14	***HHZ5-Y2-Y2***	26.8**	35.1**	13.1**
CK1	HHZ (the recipient)	24.6	9.4	16.7			9.4	24.6	16.7			16.7	24.6	9.4
CK2	PSB Rc82 (irrigated)	30.9	9.9	16.9			9.9	30.9	16.9			16.9	30.9	9.9
CK3	NSIC Rc222 (irrigated)	33.7	12.2	15.2			12.2	33.7	15.2			15.2	33.7	12.2
CK4	NSIC Rc192 (rainfed)	21.0	11.1	-			11.1	21.0	-			-	21.0	11.1
CK5	NSIC Rc184 (salinity)			21.0					21.0			21.0		
CK6	IR74371-70-1-1 (drought)	15.8	9.1	12.8			9.1	15.8	12.8			12.8	15.8	9.1

^a^ The 14 best-performing lines in each testing environment also showed their yield performance under two other environments, while *, ** and *** show the significance levels at P ≤0.05, 0.01 and 0.001 respectively, when compared with the check, HHZ. The bold and underlined numbers represent significantly higher and lower than HHZ.

The 14 best yielders under drought had a much greater yield advantage over HHZ by 95.7–146.8% or over the best check (NSIC Rc222) by 50.8‒90.2% (Table 4). Under normal conditions, five of these DT lines had significantly higher yield than HHZ by 17.9‒46.7%, two of which (HHZ17-S1-Y1 and HHZ17-D4-S3) had yield equivalent to that of the best yielding check. Under salt tress, five of these DT lines also had significantly higher yield than HHZ by 16.7‒103.6%, two of which even had significantly higher yield than the ST check (NSIC Rc184) by 28.1% and 63.8%, with only one of these DT lines showing a significantly lower yield than HHZ. Five of these DT lines resulted from selection scheme DS, three from YD, two from YS and the remaining four were from SS, YY, SY and DY, indicating that the number of best DT lines was associated with selection under either drought or normal conditions.

The 14 best ST yielders had a considerable yield advantage over HHZ by 60.5–106.0% or over the ST check (NSIC Rc184) by 27.6‒63.8% under salt stress (Table 4). Under non-stress control conditions, four of these ST lines were also good yielders equivalent to the high-yielding check and showed a yield advantage over HHZ by 17.9–46.7%, with only one line showing significantly lower yield than HHZ. Under drought stress, all except two of these ST lines had significantly higher yield than HHZ, five of which showed better DT than the best check. Surprisingly, we found that four of these ST lines resulted from selection scheme YD, three from SS or YY and two from DS or YS. Thus, the number of best ST lines was not associated with selection under salt stress.

### Promising lines and varieties developed

Looking at the results of Table 4, the 36 top performing BC_1_F_4_ ILs were derived from 28 BC_1_F_2_ selections from eight HHZ populations with 24 lines (66.7%) from three populations (13 ILs from HHZ5, 7 from HHZ17 and 4 from HHZ8). Nine lines showed significant yield improvement under all three conditions, of which HHZ17-D4-S3 and HHZ5-Y2-Y2 were among the highest yielding performers under two testing conditions. Sixteen lines showed significant yield improvement under two of the three conditions without a yield penalty under the other one, of which HHZ8-Y2-D1, HHZ5-S14-S2 and HHZ5-Y1-Y1 were the highest yielding performers under two of the testing conditions. In addition, four lines showed a huge yield improvement under one of the three conditions without a yield penalty under the other two conditions and four lines showed significant yield improvement under two of the three conditions but suffered a significant yield penalty under one of the testing conditions.

After one more round of progeny testing and two more seasons of preliminary yield trials across four testing conditions (normal, drought, salinity and submergence), many BC_1_F_5_ ILs were nominated to the NCYTs of different ecosystems in the Philippines, Pakistan, Mozambique and India from 2011 to 2015 (Table 5). Of these, nine were derived from population HHZ5 with donor OM1723, seven from HHZ8 with Phalguna as donor, four from HHZ12 with donor Teqing and only one each from populations HHZ11, HHZ15, HHZ17 and HHZ19. Interestingly, the third-round selections from a single line (HHZ8-S6-S3) under irrigated and saline conditions (HHZ8-S6-S3-Y1, HHZ8-S6-S3-Y2 and HHZ8-S6-S3-S1) resulted in three promising lines all nominated into irrigated conditions in Mozambique and rainfed dry/saline conditions (HHZ8-S6-S3-Y2). Two additional ILs, HHZ5-D7-Y3-S1 and HHZ5-S6-S3-D1, were advanced from the irrigated lowland category in NCYTs to the Multi-Adaptation Trial (MAT) under irrigated conditions in the Philippines. In the 2011–2012 dry season and coordinated by the Department of Agriculture (DA) of the Philippines, five HHZ ILs were evaluated and validated in on-farm yield trials in direct comparison with local varieties at 54 sites across the Philippines for two consecutive wet seasons (2012–2013), from which four HHZ ILs showed superior performance over the national and best farmer check varieties and were released as new varieties (GSR 5, GSR5A, GSR8 and GSR 12) for rainfed areas of the Philippines in 2013 (Table 5, S3 Table). These on-farm trials generated a huge seed demand from the farmers for wide-scale adoption. The DA and its Regional Focal Units (RFUs) embarked on a massive certified seed production drive covering more than 5,700 ha in WS2014 and produced 19,000 t of seeds of these four new varieties for targeting rainfed lowlands of the Philippines during 2015WS. Interestingly, GSR 8 and GSR 5A also showed good submergence tolerance in addition to their tolerance of drought and salinity. GSR 12 and GSR 5A also had good grain quality features such as aroma, high head-rice recovery (60‒62%) and less chalkiness. Two other HHZ ILs (HHZ5-S10-D3-Y2 and HHZ5-S12-D3-Y2) were officially released as new varieties (NIBGE I-GSR and NIBGE II-GSR) for the irrigated system in Pakistan in 2015 through the Variety Evaluation Committee (VEC) (Table 5).

**Table 5:** Six released varieties and 24 promising HHZ introgression lines nominated in the National Cooperative Yield Trials (NCYTs) of different countries.

Pedigree	Country	Year	Target environments/category	Name or likely year to be released^b^
HHZ5-S8-D3-SU1	Philippines	2012	Rainfed lowland & Aromatic	**GSR 5A**
HHZ5-S14-S2-Y2	Philippines	2012	Rainfed lowland	**GSR 5**
HHZ8-S6-S3-Y2	Philippines	2012	Rainfed lowland	**GSR 8**
HHZ12-D10-S1-D1	Philippines	2012	Rainfed lowland & Aromatic	**GSR 12**
HHZ5-S10-D3-Y2	Pakistan	2015	Irrigated	NIBGE-I-GSR
HHZ5-S12-D3-Y2	Pakistan	2015	Irrigated	NIBGE-II-GSR
HHZ17-D6-S3-D1	India	2014	Irrigated & rainfed lowland	2017
HHZ11-Y6-Y1-Y1	India	2014	Directed-seeded rice	2017
HHZ5-S9-Y3-Y1	India	2014	Directed-seeded rice	2017
HHZ5-S10-D1-D1	India	2014	Alkaline & inland saline	2017
HHZ12-S2-Y3-Y2	Mozambique	2014	PVS-irrigated^a^	2017
HHZ12-Y4-D1-Y1	Mozambique	2014	PVS-irrigated	2017
HHZ5-S12-D3-Y2	Mozambique	2014	PVS-irrigated	2017
HHZ5-S14-S2-Y1	Mozambique	2014	PVS-irrigated	2017
HHZ5-S14-S2-Y2	Mozambique	2014	PVS-irrigated	2017
HHZ5-S8-D2-S1	Mozambique	2014	PVS-irrigated	2017
HHZ5-Y3-Y1-D1	Mozambique	2014	PVS-irrigated	2017
HHZ8-S14-S3-Y2	Mozambique	2014	PVS-irrigated	2017
HHZ8-S6-S3-S1	Mozambique	2014	PVS-irrigated	2017
HHZ8-S6-S3-Y1	Mozambique	2014	PVS-irrigated	2017
HHZ8-S6-S3-Y2	Mozambique	2014	PVS-irrigated	2017
HHZ5-D7-Y3-S1	Philippines	2013	Group I irrigated	2017
HHZ5-S6-S3-D1	Philippines	2012	Group II irrigated	**NSIC Rc436**
HHZ5-S5-Y2-Y1	Philippines	2013	Special purpose-aromatic	2017
HHZ12-S2-Y3-Y2	Philippines	2013	Saline	2017
HHZ8-S6-S3-Y2	Philippines	2013	Saline	**NSIC Rc480**
HHZ12-D10-S1-D1	Philippines	2014	Saline	2017
HHZ15-S13-Y1	Philippines	2013	Upland (WS)	2017
HHZ19-S14-Y1	Philippines	2013	Upland (WS)	2017
HHZ8-S6-S3-Y2	Philippines	2013	Rainfed dry-seeded (WS)	**NSIC Rc480**

^a^ PVS = participatory varietal selection.

^b^ Bold ones are varieties officially released to farmers.

### Genotypic characterization of BC_1_F_2_ and BC_1_F_3_ selections

Because so many promising HHZ lines with good yield potential and greatly improved tolerance of one or more stresses were developed in only eight breeding populations in 6 years, we were wondering what might have happened to the genetic compositions of these selected HHZ BC progenies. Table 6 summarizes the genotyping results of the selected BC_1_F_2_ and BC_1_F_3_ progenies. The average donor introgression frequency (IF) in the 329 selected BC_1_F_2_ plants was 0.297, or 18.8% higher than the expected 0.250 in the BC_1_ progenies. However, BC progenies from different populations varied considerably for their mean donor IF. The ILs from populations HHZ9, HHZ17 and HHZ19 had mean IFs of 0.214, 0.177 and 0.168, significantly lower than the expected 0.250, whereas the ILs from populations HHZ5, HHZ12 and HHZ15 had mean IFs of 0.383, 0.436 and 0.463, significantly higher than the expectation. Furthermore, IFs of different ILs within each population varied very little with SD <0.08. This result suggested that IF appeared to be a characteristic of progenies from a specific BC population. However, testing against the expected genotypic frequency for the BC_1_F_2_ population (without any selection) showed that the genotypic frequencies of the selected BC_1_F_2_ plants from all eight populations significantly deviated from expected, with an excess of the donor homozygous genotype and much reduced frequency of the heterozygote by 68% (Table 6). When calculated across all markers of the genome, donor introgression of ILs of all eight populations showed very large variation (mean SD = 0.252), ranging from zero introgression in some genomic regions to 100% in some other regions in almost all populations. The segregation of the donor genomic segments in the selected BC_1_F_3_ ILs was consistent with the BC_1_F_2_ progenies with slightly increased frequency and variation in donor introgression, and reduced heterozygosity in ILs of most populations (Table 6).

**Table 6:** The mean genotypic and introgression frequency of selected BC_1_ introgression lines from eight HHZ populations.

Pop.	BC_1_F_2_ ^a^					BC_1_F_3_				
	N	B^b^	H	IF	SD	N	B	H	IF	SD
HHZ5	47	0.330c	0.105	0.383b	0.239	75	0.363bc	0.071	0.400bc	0.245
HHZ8	37	0.159d	0.140	0.229c	0.257	56	0.150f	0.104	0.202e	0.205
HHZ9	35	0.177d	0.074	0.214cd	0.287	62	0.300c	0.04	0.320d	0.362
HHZ11	36	0.166d	0.160	0.245c	0.130	55	0.219ef	0.091	0.264	0.180
HHZ12	43	0.397b	0.077	0.436ab	0.311	48	0.399b	0.042	0.420b	0.366
HHZ15	30	0.448a	0.029	0.463a	0.392	45	0.443a	0.007	0.448a	0.444
HHZ17	43	0.122e	0.109	0.177e	0.158	68	0.169f	0.074	0.202e	0.158
HHZ19	40	0.137de	0.062	0.168e	0.248	68	0.256e	0.036	0.184e	0.268
**Mean**	**311**	**0.244**	**0.105**	**0.297**	**0.252**	**477**	**0.273**	**0.061**	**0.304**	**0.273**

^a^ Genotypic frequency of BC_1_F_2_ plants and BC_1_F_3_ progenies that were obtained from the bulk plant tissues of the BC_1_F_3_ and BC_1_F_4_ plants of each selected plant, where B, H and IF are the mean frequency of the donor homozygotes, heterozygotes and average standard deviation of introgression frequency across the genome, respectively.

^b^ Different letters after the frequency indicate a significant difference at P ≤0.05.

When the donor introgression was compared among ILs derived from different selection schemes (Table 7), the donor introgression in the BC_1_F_2_ progenies selected under non-stress irrigated conditions, drought and salinity did not differ significantly, except that selection for high yield under the irrigated control produced progenies with significantly higher heterozygosity than those selected under drought or salinity. When the two-round selection schemes were compared, ILs from most selection schemes did not differ much in their donor introgression frequency except that the 33 ILs from DY had significantly lower introgression. Similarly, ILs from most selection schemes had similar heterozygosity except for those from selection schemes YY and YD, which had significantly higher heterozygosity.

**Table 7:** The mean genotypic and introgression frequency of BC_1_ introgression lines generated by different selection schemes from eight HHZ populations.

BC_1_F_2_ ^a^					BC_1_F_3_				
SS ^b^	N	B ^c^	H	IF±SD	SS	N	B	H	IF±SD
Y	82	0.239a	0.121a	0.290±0.068	YY	72	0.265b	0.078a	0.302±0.076
D	109	0.250a	0.097b	0.302±0.063	DY	33	0.137d	0.033b	0.154±0.052
S	120	0.234a	0.087b	0.277±0.056	SY	57	0.217c	0.050b	0.242±0.048
					YD	59	0.260b	0.074a	0.297±0.053
					DD	24	0.260b	0.048b	0.284±0.033
					SD	20	0.301a	0.049b	0.325±0.035
					YS	21	0.248bc	0.047b	0.271±0.048
					DS	151	0.192c	0.041b	0.212±0.046
					SS	54	0.247bc	0.051b	0.273±0.068
**Mean**	**311**	**0.244**	**0.105**	**0.297±0.062**		**491**	**0.273**	**0.061**	**0.304±0.052**

^a^ Genotypic frequency of the BC_1_F_2_ plants or BC_1_F_3_ progenies was obtained from the bulk plant tissues of their BC_1_F_3_ or BC_1_F_4_ progenies of each selected plant, where B, H and IF are the mean frequency of the donor homozygote, heterozygote and donor introgression, respectively, calculated from all genotyped SSR markers.

^b^ SS = selection scheme, with Y, D and S representing selections for high yield under normal conditions, drought stress and salinity stress in the first round of selection in the BC_1_F_2_ generation, while YY, DY, SY, and SS represent nine selection schemes of two rounds of selection with the first letter representing the target trait (or testing environment) of the first round of selection in the BC_1_F_2_ generation, and the second letter representing the target trait of the second round of selection (or testing environment) in the BC_1_F_3_ generation.

^c^ Different letters after the frequency indicate a significant difference at P ≤0.05.

## Discussion

The low rice productivity in most rainfed areas is known to be associated with multiple abiotic stresses, but most breeding programs for rainfed systems are designed and practiced for solving problems of single abiotic stresses. In our previous efforts, we have demonstrated that BC breeding plus strong phenotypic selection is a powerful way to exploit the hidden diversity in the primary gene pool for improving single abiotic stress tolerance [7–9, 11, 15–17]. In fact, one of our DT BC_2_ ILs, DGI75, derived from the cross between IR64 (recipient) and BR24 (donor), was among the two highest yield performers in 22 trials under non-stress, moderate drought and severe drought conditions in the natural rainfed shallow lowlands in northern India [18]. In this article, we reported a modified BC breeding procedure for improving multiple complex traits, which resulted in the release and up-scaling of four new rice varieties for rainfed areas of the Philippines with superior yield potential and good tolerance of drought and salinity, two new varieties for the irrigated areas of Pakistan, plus many promising ones in the pipeline to be released from only eight BC populations in 6 years. Compared with most conventional breeding programs, our breeding approach is highly efficient. Several aspects in our BC breeding procedure that are relevant to improved selection efficiency and overall genetic gain for improving multiple complex traits merit further discussion.

### Parental selection

When compared with results from our previous study (Ali et al. 2006), results from this study indicated that selecting appropriate recurrent parents is the key for success in improving complex traits in a BC breeding program. HHZ was therefore chosen as the recipient of our BC breeding program because it is the most widely grown inbred variety in China with high yield potential and many desirable features such as excellent plant type and grain quality. When evaluated under favorable irrigated conditions, it also showed high yield in more than 13 countries across Asia and Africa in our GSR project (the year 1 report of GSR II), indicating its wide adaptability. In classical BC breeding, most breeders tend to use local best varieties as the recipients of their BC breeding programs, which may or may not be suitable ones. The obvious reason is that a widely adaptable variety must have key adaptive traits and pathways to most environments, in addition to its high yield potential, and it requires minimum numbers of additional alleles for improving specific target traits. Apparently, HHZ meets the requirements as an excellent recipient. Even with one generation of backcrossing, the influence of the HHZ genetic background on the overall performance of its BC progenies was enormous, as most HHZ ILs resembled HHZ phenotypically with short stature, relatively small and erect leaves and compact panicles. However, we noted that HHZ and most of its progenies were susceptible to Tungro viruses, and this seemed difficult to change. Thus, the recipients of BC breeding programs for complex trait improvement should be widely adaptable superior commercial lines with minimum weaknesses and it is more desirable for a BC breeding program to have two to three recipients with different plant types if the breeding is aiming at broad and diverse target environments.

We found that more promising ILs were selected from populations HHZ5 (OM1723) and HHZ17 (CDR22) than others, indicating that donors did make differences in breeding efficiency, when defined as the number of new varieties and promising lines developed per breeding population, and OM1723 and CDR22 have favorable alleles for the target traits at more loci complementary to HHZ. This was expected because OM1723 and CDR22 were more distantly related to HHZ than other donors based on our SSR data. This result suggests that BC breeding for improving complex traits should not use closely related donors. Nevertheless, we did not make any attempt in donor selection because experiences in our previous breeding efforts indicated that donor selection based on target phenotypes was a poor way to identify superior donors for improving complex traits. In fact, the eight donors mostly represent elite materials under irrigated conditions and none of them has good tolerance of drought and salinity, and transgressive progenies, though fewer, were identified in all other populations, indicating that all donors have favorable alleles for the target traits. Since most favorable alleles for the target traits (HY, DT and ST) from different donors are unlikely the same ones, introgression of the favorable alleles from different donors into the recipient was expected to broaden the genetic diversity for the target traits in the HHZ ILs. Again, this study provided another piece of strong evidence for the presence of rich hidden genetic diversity for complex traits in the primary gene pool of rice.

### Differences in different selection schemes

In this study, the responses of the BC progenies to phenotypic selection were quite complex. When the nine selection schemes were compared, most selection schemes produced approximately equal numbers of high and low yielders under irrigated and saline conditions. This did not suggest ineffectiveness of the first two rounds of selection for improving yield and ST, nor did it indicate that different selection schemes had the same breeding efficiency because the selected progenies were not compared directly with unselected ones and the two rounds of selection for the same traits practiced in different seasons (here, the wet and dry seasons) may not act on the same suites of alleles and pathways. We noted that all selection schemes resulted in significantly more DT lines. Particularly, 12 of the top 14 best performing lines under normal or saline conditions showed significantly improved yield under drought (Table 4), but the opposite was not true. This type of partial association between yield and DT has been reported in rice [16, 19], wheat [20], maize [21–23] and common beans [24], but their underlying genetic mechanism remains largely unknown. Also, it was intriguing to note that most ILs selected under salt stress had significantly improved DT but not necessarily ST. The correlated response of salt-selected ILs to drought could be explained by the common physiological mechanisms and genetic basis underlying both DT and ST in rice [25]. Interestingly, we found that the first two rounds of selection for ST at the seedling stage did not result in more BC progenies that performed better in the replicated trials under the whole growth-duration salt stress of the field conditions, which could, at least partially, be attributed to the differences between genetic and molecular mechanisms underlying ST at the seedling stage and that at the reproductive stage [26. 27], and partially to the possible mineral deficiencies (Zn, P) and/or toxicities (Fe, Al, organic acids) that are commonly compounded with salinity in coastal salinized fields [2]. Thus, this result clearly indicated that selection for abiotic stress tolerance such as DT and ST should be best practiced in the target environments at the right developmental stage. Nevertheless, the selection process in our BC breeding procedure with strong single-plant selection in the early segregating generation followed by one or two rounds of progeny testing across both stress (multiple) and non-stress environments has two unique advantages: (1) the high selection intensity, particularly under the stress conditions in the first round of selection, could quickly remove most progenies that do not have target stress tolerance; and (2) the second-round progeny testing of all selected progenies across multiple testing environments would not only verify the effectiveness of the first-round single-plant selection for single target traits, but would also allow full exploitation of the residual genetic variation in the progenies selected for a single trait for other target traits under multiple testing environments. In fact, all selected HHZ ILs had gone through third-round progeny testing across drought, salinity, submergence and non-stress conditions and had been evaluated for grain quality parameters and resistance to several biotic stresses such as rice blast, bacterial blight and Tungro viruses. Again, considerable variation remains in the HHZ ILs for all measured traits, providing valuable data for gene/QTL discovery using the HHZ ILs and DNA markers.

### Donor introgression characteristics of stress-selected BC progenies

Using DNA markers, we were able to reveal some interesting aspects of donor introgression in different BC progenies of rice and their responses to strong phenotypic selection. First, the proportion of donor introgression in a BC population is a unique feature of specific crosses or recipient/donor combinations, reflected by small variation in introgression among individual lines from the same populations, but huge differences among lines from different populations (Table 6). In other words, introgression or segregation in BC progenies of rice line crosses was characteristic of specific crosses, which may or may not follow Mendelian expectations. Selection did not appear to be responsible because no significant differences in IF were detected among lines from different selection schemes of the same populations. Since all parental lines belong to *indica* subspecies, the distorted segregation in the eight populations should be at a minimum. Then, a question arises regarding what mechanisms are responsible for the observed segregation distortion in specific BC populations, which should be addressed in the future. Second, we observed generally reduced heterozygosity in the BC progenies selected in both rounds of selection. In particular, more dramatically reduced heterozygosity was associated with selection for DT and ST than selection for yield under non-stress conditions. This type of greatly reduced heterozygosity was also observed in the BC progenies selected for tolerance of drought, salinity and submergence [25, 28, 29]. This explained why our breeding strategy took a short time in varietal development compared with conventional bi-parental pedigree breeding, which is normally practiced for high yield under non-stress irrigated conditions and takes at least 8‒10 years from cross making to farmers’ fields. Empirically, it is generally true that breeders can easily identify many “superior” high-yielding plants in the early generations of their pedigree breeding populations under non-stress conditions, but these “superior” plants often segregate markedly for an extended period of time up to F_10_ or higher generations, and many of their desirable traits originally selected are disappearing as the progenies reach complete homozygosity. Obviously, the superior performance in vigor and yield of these early-generation progenies is due largely to heterosis. To overcome this, it was suggested that selection for yield potential should be delayed to later generations [30]. In this regard, screening for abiotic stress tolerance practiced in our BC breeding program appeared to allow simultaneous improvement of one or more abiotic stress tolerances as well as to achieve quicker homozygosity in early breeding generations. However, it remains a major challenge to understand what genetic and molecular mechanisms are responsible for the stress-induced quick homozygotization of early-generation breeding materials.

It should be pointed out that what was presented in this study was part of the GSR breeding technology, which can be relatively easily adopted by breeding programs in developing countries to develop varieties suitable for any target traits in different rice ecosystems. However, the real power of the GSR breeding strategy is the full integration of trait-specific ILs with the new genomic technologies [28]. Using the same BC breeding procedure, we have successfully converted HHZ into 2,000+ ILs with significantly improved tolerance of more abiotic stresses (drought, salinity, submergence, etc.) within 5 years, including the 496 HHZ BC_1_F_5_ ILs presented above, 832 HHZ BC_1_F_5_ ILs from another eight BC_1_ populations and 1,000+ BC_2_ ILs [31]. These HHZ ILs formed the material platform for identifying genes/QTLs controlling the target and non-target traits and for highly efficient development of new GSR varieties by designed QTL pyramiding and genomics-based recurrent selection of the GSR breeding technology [28]. Currently, HHZ and the eight donors have been completely sequenced. The 496 HHZ BC_1_F_5_ ILs have been genotyped by sequencing and phenotyped for yield traits under different stress (drought, low input, salinity and cold) conditions across many locations in Asia. They have also been evaluated for quality parameters and resistance to multiple races of rice blast and bacterial blight pathogens. Analyses of this huge amount of data are in progress to identify genes/alleles underlying the target (HY, DT and ST) and non-target (resistance to biotic stresses, quality traits, cold tolerance, etc.) traits, which will be published soon. The genetic and phenotypic information of the HHZ ILs is being used for more efficiently developing superior varieties with better performance by designed QTL pyramiding and marker-assisted recurrent selection [28].

## Conclusions

In conclusion, we reported here a highly efficient BC breeding procedure for improving multiple complex traits. Using this approach, we were able to develop large numbers of ILs with significantly higher yield and tolerance of drought and salinity from eight BC populations in 6 years. Six of these ILs were released as new varieties for the rainfed and irrigated areas of the Philippines and Pakistan. Genetic characterization of selected BC_1_F_2_ plants and BC_1_F_3_ lines by DNA markers reveals three interesting aspects of donor introgression in rice BC populations: (1) donor introgression varied considerably across different crosses, (2) donor introgression in different genomic regions varied considerably across the genome in the selected ILs resulting primarily from strong selection for target traits, and (3) greatly reduced heterozygosity was observed in the selected BC progenies, particularly for drought- and salinity-selected ones. Thus, applying strong phenotypic selection under severe abiotic stresses has major advantages by not only improving one or more abiotic stress tolerances, but also being able to achieve quicker homozygosity in early breeding generations. This breeding procedure can be relatively easily adopted by small breeding programs in developing countries to develop varieties suitable for complex target traits in different rice ecosystems. The large set of trait-specific ILs with significantly improved tolerance of more abiotic stresses formed the material platform for large-scale discovery of genes/QTLs underlying the target and non-target traits and for highly efficient development of new Green Super Rice varieties by designed QTL pyramiding and genomics-based recurrent selection.

## Supporting information

S1 Table Weather conditions prevalent during the breeding cycle across dry and wet season from 2008 to 2013 at IRRI, Los Baños, Philippines. (DOCX)

S2 Table The original data for grain yield and its related traits of the 496 ILs selected by nine selection schemes under three environments. (XLSX)

S3 Table The performance of four promising released GSR varieties in the Philippines across 54 sites in rainfed and irrigated lowlands. (DOCX)
