# Supplementary material for: Harnessing the hidden genetic diversity for improving multiple abiotic stress tolerance in rice (Oryza sativa L.)
Source: PLoS One. 2017 Mar 9;12(3):e0172515. doi: 10.1371/journal.pone.0172515 (PMC5344367; doi:10.1371/journal.pone.0172515)
Supplement: S1 Table — (DOCX) [file pone.0172515.s001.docx]

| **S1 Table Weather conditions prevalent during the breeding cycle across dry and wet season from 2008 to 2013 at IRRI, Los Baños, Philippines** | | | | | | | | | | | | | | | | | | | |  |
| --- | --- | --- | --- | --- | --- | --- | --- | --- | --- | --- | --- | --- | --- | --- | --- | --- | --- | --- | --- | --- |
| **Season** | **Month** | **2008** | | | **2009** | | | **2010** | | | **2011** | | | **2012** | | | **2013** | | | |
|  |  | **Rain Fall** | **Mean Temp** | **RH** | **Rain Fall** | **Mean Temp** | **RH** | **Rain Fall** | **Mean Temp** | **RH** | **Rain Fall** | **Mean Temp** | **RH** | **Rain Fall** | **Mean Temp** | **RH** | **Rain Fall** | **Mean Temp** | | **RH** |
|  |  |  |  |  |  |  |  |  |  |  |  |  |  |  |  |  |  |  |  |  |
|  |  | **mm** | **^o^C** | **%** | **mm** | **^o^C** | **%** | **mm** | **^o^C** | **%** | **mm** | **^o^C** | **%** | **mm** | **^o^C** | **%** | **mm** | **^o^C** | | **%** |
| Dry Season | Jan | 172.6 | 25.8 | 85.8 | 113.3 | 24.9 | 83.5 | 14.6 | 26.3 | 88.8 | 245.1 | 27.8 | 98.4 | 83.5 | 26.3 | 87.4 | 121.8 | 25.6 | | 84.9 |
|  | Feb | 60.2 | 25.3 | 85.9 | 51.6 | 26.4 | 85.2 | 2.9 | 26.3 | 87.0 | 122.6 | 25.4 | 86.7 | 87.0 | 26.2 | 87.4 | 146.0 | 26.1 | | 84.8 |
|  | Mar | 7.1 | 26.4 | 78.8 | 123.7 | 27.4 | 86.8 | 26.0 | 27.0 | 84.8 | 68.0 | 25.9 | 86.3 | 111.0 | 26.4 | 86.9 | 53.6 | 27.3 | | 82.7 |
|  | Apr | 53.5 | 28.3 | 82.1 | 341.1 | 28.0 | 88.0 | 47.7 | 28.5 | 80.8 | 7.6 | 26.9 | 81.6 | 35.0 | 28.3 | 82.3 | 11.6 | 29.3 | | 77.9 |
| Wet Season | Jun | 294.4 | 28.3 | 82.3 | 292.1 | 28.3 | 84.8 | 199.9 | 29.1 | 84.0 | 423.6 | 28.0 | 87.1 | 74.5 | 28.5 | 83.7 | 270.3 | 28.9 | | 82.8 |
|  | Jul | 162.4 | 28.0 | 85.1 | 365.7 | 28.2 | 87.2 | 683.7 | 28.2 | 87.1 | 299.6 | 27.5 | 87.6 | 458.1 | 27.8 | 87.0 | 335.2 | 28.1 | | 86.2 |
|  | Aug | 226.7 | 27.5 | 87.6 | 140.9 | 28.5 | 86.5 | 215.0 | 28.2 | 89.5 | 297.8 | 27.7 | 87.7 | 464.8 | 27.6 | 85.8 | 430.2 | 27.8 | | 87.1 |
|  | Sep | 131.1 | 28.0 | 85.9 | 233.8 | 27.8 | 90.0 | 124.2 | 28.0 | 88.8 | 222.5 | 27.8 | 87.3 | 146.9 | 28.0 | 88.1 | 406.1 | 27.6 | | 88.7 |
| Source: Climate Unit, Crop and Environmental Sciences Division, IRRI | | | | | | |  |  |  | Temp=Temperature; RH=Relative Humidity | | | | |  |  |  |  | |  |
|  | | | | | | | | | | | | | | | | | | |  |  |
