# Supplementary material for: Harnessing the hidden genetic diversity for improving multiple abiotic stress tolerance in rice (Oryza sativa L.)
Source: PLoS One. 2017 Mar 9;12(3):e0172515. doi: 10.1371/journal.pone.0172515 (PMC5344367; doi:10.1371/journal.pone.0172515)
Supplement: S3 Table — (DOCX) [file pone.0172515.s003.docx]

| **S3 Table Performance of 4 promising GSR released varieties in the Philippines across 54 sites* in rainfed and irrigated lowlands** | | | | |
| --- | --- | --- | --- | --- |
| **New Designation** | **Old designation** | **Overall Mean Grain Yield (t/ha)** | **% Yield advantage over average of best checks** | **Total sites studied** |
| GSR IR1-5-S14-S2-Y2 (GSR 5) | HHZ5-S14-S2-Y2 | 4.98 | 10.67 | 54 |
| GSR IR1-5-S8-D3-SU1 (GSR5A) | HHZ5-S8-D3-SU1 | 4.82 | 7.11 | 38 |
| GSR IR1-8-S6-S3-Y2 (GSR8) | HHZ8-S6-S3-Y2 | 5.07 | 12.67 | 54 |
| GSR IR1-12-D10-S1-D1 (GSR12) | HHZ12-D10-S1-D1 | 5.12 | 13.78 | 54 |
| National**/Farmer's Best Check Varieties |  | 4.50 | - | 54 |
| *WS2012=21 sites and WS2013=33 sites; Department of Agriculture and IRRI, Philippines jointly organized trials in farmer's field; **National best checks used NSIC Rc222,214,160,184,188,238,224,192,& 240;PSB Rc4,10,82,& 138; Farmer Varieties: Sinampaguita, Matatag and Blonde | | | | |
